# The Dignity Project Framework: An extreme citizen science framework in occupational therapy and rehabilitation research

**DOI:** 10.1111/1440-1630.12847

**Published:** 2022-11-12

**Authors:** Kelsey Chapman, Angel Dixon, Kevin Cocks, Carolyn Ehrlich, Elizabeth Kendall

**Affiliations:** ^1^ Hopkins Centre Griffith University Nathan Queensland Australia; ^2^ Griffith University Southport Queensland Australia

**Keywords:** citizen science, consumer engagement, co‐researchers with disability, dignified research, dignity, disability studies, inclusive research

## Abstract

**Introduction:**

Engaging citizens and patients as research partners is receiving increasing emphasis across disciplines, because citizens are untapped resources for solving complex problems. Occupational therapists are engaging in inclusive research, but not always in equitable partnership. Moving beyond inclusive research to a dignified framework for research prioritises lived experience and human rights in health research.

**Methods:**

Using nominal group technique over a series of three working group meetings, eight experts, including three with lived experience of disability and research, prioritised principles and steps for conducting dignified rehabilitation research in partnership with citizens with disability.

**Findings:**

Embedding transparency, accessibility and inclusion, dignified language, and authenticity throughout research were integral to maintaining dignity and safety for citizens with disability engaged in research. The Dignity Project Framework encompasses five phases, namely, (1) vision, (2) uncover, (3) discuss, (4) critical reflection, and (5) change, which address the prominent criticisms of the disability community about research and embed the principles of importance into research practice.

**Conclusion:**

The framework builds on inclusive research frameworks to a human rights‐based, dignified framework for extreme citizen science. Grounding disability in contemporary conceptualisations and providing a method for democratising knowledge production provide occupational therapists with a method for dignified partnership with citizens with disability.

## 1 INTRODUCTION

Citizens are a potentially vast and relatively untapped resource for solving complex societal problems (Rowbotham et al., 2019). When citizens participate and are engaged in research, they bring real‐world insight and perspective, while simultaneously increasing recruitment and retention of other citizens (Greenhalgh et al., 2019). Unsurprisingly, there is increasing interest and emphasis on citizen engagement in research across government, health services, industry, and academia (Bonny et al., 2009). The legislative imperative for engaging citizens in health research is well established under “Standard 2: Partnering with consumers” in the Australian Commission on Safety and Quality in Healthcare (2017) (Ehrlich et al., 2020). Recent implementation of consumer and community advisory panels by both the NHMRC and MRFF further highlights the importance for health researchers to build capacity and capability to engage consumers in research. Within the fields of health research, occupational therapists are well placed to support citizen engagement, due to their knowledge of person‐centred practice and close partnership with patients to achieve positive health outcomes (Layton, 2014; Pereira et al., 2020). However, “consumer engagement in occupational therapy research may be in its infancy” (Cox et al., 2021).

The rise of the expert consumer and the increasing emphasis placed on the importance of citizen voices in health research (Layton, 2014) have led to a flood of values‐based frameworks, principles, and methods for “engaging, involving, including, and partnering with consumers” (Ehrlich et al., 2020, p. 5). Best practice citizen engagement methods include co‐design (Robert et al., 2015; Steen et al., 2011), co‐production (Batalden et al., 2016; Whitaker, 1908), and co‐creation (Frow et al., 2016; Hardyman et al., 2015; Voorberg et al., 2015) and are often centred on principles for inclusive research (Frankena et al., 2019; Layton, 2014; Pereira et al., 2020), many of which are currently used by occupational therapists. The importance of inclusive research is particularly salient to citizens with disability, a key cohort population in occupational therapy, for whom research has historically been problematic and often tokenistic (Ehrlich et al., 2020; Goodley, 2017; Pena et al., 2016; Slattery et al., 2020). Frameworks and guidelines for the inclusion of citizens with disability in health research attempt to address exclusionary barriers to increase engagement. The Disability Inclusive Research Collaboration released its *Quality Framework for Inclusive Research* (2012), which describes 10 principles for conducting emancipatory and participatory science with citizens with disability. Many of these same principles such as co‐production, accessible methods, reciprocal benefit, and priorities driven by the community are echoed in O'Brien et al. (2014) consensus statement for conducting inclusive health research with citizens with intellectual disability. Whereas occupational therapy research embraces the principles and frameworks for inclusive research with citizens with disability, research is often driven by professional or clinical researchers in consultation with citizens with disability, who may be included for member checking or rigour, rather than engaged in meaningful partnership (Cox et al., 2021).

Empowering citizens with disability to use their lived experience knowledge, as scientific citizens to impact change, can have positive research outcomes for individuals, occupational therapy researchers, and whole communities, but only when done authentically and with dignity (Chesser et al., 2020; King et al., 2016; Sheats et al., 2013). Inclusive research frameworks provide strong foundations for representative health research for citizens with disability. However, even best practice inclusive research can revert to the normative dynamics that govern traditional research (Albert et al., 2021; Hidalgo et al., 2021; Paleco et al., 2021). Community exploitation, inequitable involvement, and reverting to tokenistic consultation of citizens with disability can occur, even with the best intentions (English et al., 2018).

There is a recent call among specialists in Diversity, Equity, and Inclusion (DEI) to move away from a focus on inclusion towards a human rights‐based focus on dignity (Davis, 2021). Dignity, which includes principles like inclusion and accessibility, acknowledges the inherent value of every individual, “regardless of positional power and privilege” (Davis, 2021). Ensuring citizens with disability are engaged in ways that are dignified acknowledges the tokenism and exploitation historical to health and occupational therapy while affirming the importance of individual lived experience, personhood, and indigenous knowledge (Davis, 2021).

Extreme citizen science, a method typically used for citizen engagement in the natural sciences, has the potential to offer an ultra‐inclusive, dignified approach to research (English et al., 2018; Haklay, 2013; Stevens et al., 2014). Grounded in Alan Irwin's original conceptualisation of citizen science, in which research is done for and by citizens, with an emphasis on co‐design and co‐creation of knowledge, extreme citizen science has the potential to enact meaningful citizen engagement outcomes that produce implementable change in health research fields, like occupational therapy (Arstein‐Kerslake et al., 2020; Borda et al., 2019; Irwin, 1995; Strasser et al., 2019). Extreme citizen science gives voice to diverse groups and new perspectives, especially among populations that are traditionally silenced in and by research (Green, 2016).

Extreme citizen science is defined as a collaborative science—one in which citizens are engaged in defining scientific problems and in collecting and analysing data (Haklay, 2013). Citizens lead and drive extreme citizen science research in equal partnership with clinical and occupational therapy researchers (Borda et al., 2019; Pejovic & Skarlatidou, 2019; Strasser et al., 2019). Extreme citizen science embeds citizen scientists with indigenous knowledge of lived experience into a research team, preferably in senior roles (Arstein‐Kerslake et al., 2020; Greenhalgh et al., 2019; Pejovic & Skarlatidou, 2019), which supports novel research and empowers communities by increasing science literacy and decision‐making power (English et al., 2018). Thus, extreme citizen science is a “bottom‐up practice used to empower people by supporting them, via processes and technological tools, to find solutions for local problems” (Pejovic & Skarlatidou, 2019, p. 251).

Despite the potential for extreme citizen science to offer health and occupational therapy researchers a method for dignified partnership with citizens with disability, there are a dearth of research frameworks that centre on the lens of dignity. The Dignity Project, a research and advocacy initiative of Griffith University commenced in 2016 in response to research, revealed citizens with acquired impairment often experienced violations of dignity and suffered inadvertent harm through systems of care. The original aim of the Dignity Project was to collect stories and perspectives from citizens with disability to better inform clinical practice in rehabilitation, a field in which occupational therapists are often engaged. However, the lack of clarity and consensus on the ways to conduct dignified rehabilitation research in partnership with citizens with disability prompted a shift. The shift in focus centred on developing a framework to support dignified, extreme citizen science in rehabilitation research, in partnership with citizens with disability. The development of the framework is presented in the remainder of this article.

## 2 METHODS

The Dignity Project Framework for extreme citizen science was developed through adherence to the principles of extreme citizen science (Arstein‐Kerslake et al., 2020; Greenhalgh et al., 2019; Pejovic & Skarlatidou, 2019), most importantly, embedding researchers with lived experience in senior roles. The framework development was part of a larger study approved by the Metro South Hospital and Health Service Human Research Ethics Committee (HREC/2019/QMS/58929). The ethical principles of the Declaration of Helsinki were followed. Informed consent was obtained from all working group members.

### 2.1 Participants in the framework development working group

The research team (the authors) sought to develop a framework for dignified extreme citizen science to support a larger research initiative. Authors were already a mix of academics (senior and early career) with family experience of disability and impairment and citizen researchers with lived experience of disability. The authors convened a working group, as described below, with an additional three experts who had lived experience of different neurological impairment from that of the authors and/or clinical experience working in occupational therapy or rehabilitation. Although the authors organised and participated in the working group, they did not lead the discussion and appointed another facilitator from among the eight working group members to drive each of the three sessions.

Each member of the working group has either (1) extensive experience in consumer engagement research, specifically participatory inclusive research, co‐design, and citizen science; (2) rehabilitation clinical experience or theoretical experience related to disability studies; or (3) lived experience of disability and experience in research. All working group members were based in Queensland, Australia, during the working group sessions.

Three members of the working group were senior researchers with doctoral qualifications in disability and rehabilitation, with strong track records (10+ years) in emancipatory and participatory forms of research. Two were also clinical researchers with extensive health consumer engagement experience in rehabilitation. Three members were citizens with lived experience of diverse neurological impairment and engagement in research, including involvement in critical disability reform in Australia. One member was a doctoral candidate, specialising in dignified experiences of people with disability in interaction with services and systems (Chapman et al., 2022), and was specifically selected to provide theoretical insight into dignity theory as a lens for the framework (Killmister, 2020). Working group members included three males and five females with a diverse age range, the youngest being 29 at the time and the oldest being 60+.

### 2.2 Framework development process

The framework was developed over a 6‐month period from 2019 to 2020, in which the working group met three times, for an average of 2 h per session. All eight members of the working group attended all sessions. Sessions were a hybrid of in‐person and virtual both for accessibility and due to public health policies related to COVID‐19. All sessions were audio recorded and transcribed. Open forum discussion and modified nominal group technique (NGT) were used to develop consensus about principles of importance and framework tasks while allowing for balanced participation and minimising risk of a dominant participation influencing discussion (McMillan et al., 2016).

During the first session, experts identified critical priorities for dignified research when done in partnership with people with disability. Existing frameworks for consumer engagement and relevant citizen science literature were identified and discussed. Barriers to dignified rehabilitation research with people with disability were identified and discussed. Following discussion, four principles of importance were agreed upon, as discussed in Section 3.

The second session revisited the principles of importance to verify consensus. Experts were asked about implementation or application of the principles to research, including the practicalities and challenges of doing research with people with disability. Frameworks for citizen science and consumer engagement were revisited, and a first draft of the Dignity Project Framework was developed.

The third and final session once again revisited the principles of importance to verify ongoing consensus and then opened discussion on the Dignity Project Framework. Following extensive discussion, consensus was reached on the elements of the framework, including potential elements for implementation.

## 3 RESULTS

### 3.1 Principles of importance

Four principles of importance for conducting dignified rehabilitation research with people with disability as researcher partners were agreed upon by the working group: (1) grounding research in a human rights conceptualisation of disability; (2) eliminating barriers to participation—intersectionality and authenticity; (3) diversity in engagement—accessibility and inclusion; and (4) transparent ways of working.

#### 3.1.1 Principle 1: Grounding research in a human rights conceptualisation of disability

The working group identified, particularly the experts with lived experience of disability, that dignified partnership in research should be predicated on grounding research language and values in a contemporary, human rights‐based conceptualisation of disability. The United Nations Convention on the Rights of Persons with Disability (CRPD) provides a foundation for a widely accepted conceptualisation of disability. The CRPD states that “disability results from the interaction between persons with impairments and attitudinal and environmental barriers that hinders full and effective participation in society on an equal basis with others” (United Nations, 2006, p. 2). The CRPD conceptualisation of disability is affirmed internationally and locally in Australia by disabled persons organisations (DPOs), government, non‐government organisations, and disability rights activists. The working group experts recognised that that language plays a critical role in forming identities and prioritised the use of person first language (e.g. people/citizens with disability), which privileges the individual before the impairment or disability, if relevant. Language such as “suffer”, “afflicted”, “wheelchair bound”, and other terminology that patronises the human experience as “inspirational” were rejected, as were pejoratives from diagnostic language and medical terminology.

#### 3.1.2 Principle 2: Eliminating barriers to participation—intersectionality and authenticity

Engaging citizen scientists with diverse impairments in authentic ways emerged as an important principle. The working group agreed that disability is not a homogeneous experience, even among citizens with the same impairment. Lived experience is diverse, as are individual identities. The working group identified that intersecting perspectives and elements that inform self‐identity, referred to as intersectionality, can impact on lived experience of disability. It is important to embed diversity of experience on both the research team and in the participant pool.

Authenticity related to intersectionality and partnership was a key concern for the working group, particularly in relation to engaging with citizen scientists with disability. Citizen scientists can be remunerated and valued in different ways, with varying degrees of success. Some citizen scientists, as discussed by the working group, are not remunerated, remunerated through gift vouchers or small stipends, depending on feasibility. The working group identified that transitioning from volunteerism towards the professionalisation of lived experience required remuneration at market rate, although it may be difficult to achieve.

Remuneration discussions went beyond traditional forms of currency to include the currency of academia, including authorship on publications, co‐presentation at conferences, and establishment as a co‐investigator on future grant applications. At a minimum, reciprocity through mutual benefit should be agreed upon and achieved. Reciprocity may include “a meaningful stake in data ownership” (Chesser et al., 2020) and the production of change management that most benefit the community.

#### 3.1.3 Principle 3: Diversity in engagement—accessibility and inclusion

Accessibility and inclusion as a means for promoting dignified, authentic research partnership was a key principle for the working group and is well supported in contemporaneous literature. The typical methods for conducting rehabilitation research, even citizen science research, can be problematic for citizens with disability, who experience higher levels of economic inequality, may lack appropriate transportation, may experience time constraints, and could be unfamiliar with certain scientific methods (Chesser et al., 2020; Evans et al., 2005). Discussions about inclusion prompted the working group to consider the importance of flexible and atypical working patterns as important for enabling the contribution of diverse perspectives and knowledge, while accommodating the practical realities of living with impairment. Hosting meetings online, providing closed captions, shorter meetings, and working from home were all agreed upon as appropriate and preferred ways of working to include as diverse a group of citizens with lived experience of disability as possible.

Democratic means for knowledge production were also discussed. Consensus was reached about the importance of accessibility to ensure that citizens with disability engaged as equal partners in research. Ensuring online environments, content platforms, and data collection mechanisms can be accessed and understood by citizens with diverse impairment is important for enabling a safe space for participation and engagement. The working group agreed that sufficient time is needed to understand the needs of citizens with diverse impairment during data collection and analysis. Prior to commencing data collection, user testing or pilot testing methods through practice sessions or evaluations can increase access and inclusion of research methods. User testing can be undertaken by members of the target sample or by a third‐party reviewer, like the Centre for Accessibility, to develop methods that work for the largest diversity of citizens.

#### 3.1.4 Principle 4: Transparent ways of working

Transparency emerged as a key enabler to dignified rehabilitation research. The legacy of tokenism and objectification of citizens with disability in research was ever present in discussions. Maintaining good intentions was insufficient to ensure that citizen scientists with disability would feel comfortable and safe. Transparency and transparent ways of working was identified by the working group as a critical mechanism for eliminating tokenism, gate keeping, and traditional power dynamics of research when working with people with disability. Setting expectations, committing to governance processes, and enabling capacity building for both citizen scientists with disability and professional researchers were identified as mechanisms for embedding transparent ways of working. The working group emphasised the importance of developing operational documents and negotiated commitments that could be made public in order to ensure transparency was embedded throughout the life cycle of research projects.

Critical reflection and reflexive examination were discussed as mechanisms for accountability and transparency. Self‐reflection can be important when working with citizen scientists with disability, because it assists researchers to rethink their research approaches, their position and privilege during the research process, and the broader practical application context of the research method. As supported in contemporaneous literature and identified by the working group, reflection can support questioning and challenging assumptions and realise the potential of generating new knowledge (Salih & Butler, 2004; Shildrick, 2020). Critical reflection, in an iterative and focused way, was agreed to by the working group as a mechanism for resisting reversion to the normative traditions of research and for maintaining transparent, rigorous commitment to dignified research partnership with citizens with disability.

### 3.2 The Dignity Project Framework for extreme citizen science in rehabilitation research

**FIGURE 1:**
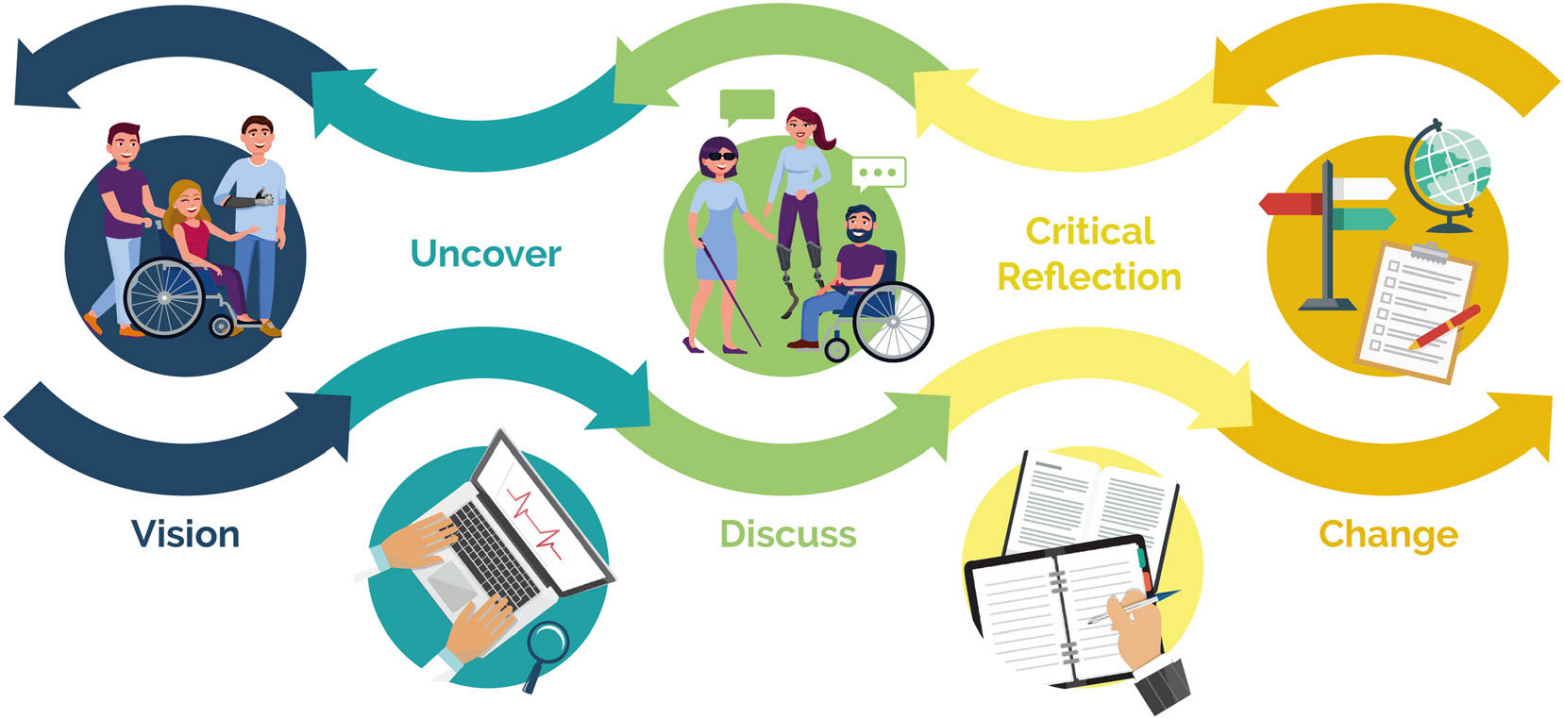
The Dignity Project Framework for extreme citizen science in rehabilitation research

In Sessions 2 and 3, the working group revisited the principles of importance and discussed how to practically embed them into rehabilitation research in partnership with citizens with disability. The resulting framework for extreme citizen science in rehabilitation research includes five phases: vision, uncover, discussion, critical reflection, and change, as illustrated in Figure 1.

#### 3.2.1 Vision

The purpose of the vision phase is to establish a holistic approach to dignified citizen science research, in which values, language, methods, and citizen involvement are envisioned, negotiated, and selected by all members of the research team. A key priority of the vision stage is to ensure that citizen scientists with disability are embedded as co‐investigators, in ways that are authentic, inclusive, and transparent to safeguard the perspectives of lived experience. A shared vision should underpin extreme citizen science projects to prevent reversion to traditional normative dynamics of tokenistic and oppressive research for citizen with disability. Transparency and authenticity in this phase came at a cost to efficiency and time.

The tasks for consideration during the vision phase, include, but are not limited to:

1. Negotiating roles, remuneration, reciprocity, and expectations for the entire life cycle of research.
2. Creating shared context, which includes capacity building and training for both citizen scientists with disability and academic/clinical researchers on the team.
3. Develop operational documents, including all negotiated values and commitments. Publicly publishing documents online via a website serves as a mechanism for promoting research transparency, accessibility, and accountability to the wider community.
4. Address flexible work patterns and ways of working that eliminate barriers to engagement of co‐researchers with disability.

#### 3.2.2 Uncover

‘Uncover’ describes the phase related to recruitment and data collection. The word ‘uncover’ was intentionally chosen by the working group because research done in partnership with citizens with disability about their lived experience often does not discover new information, but rather uncovers stories and perspectives that have long been silenced or marginalised. Safe spaces must be created for citizens with disability. The challenge is to uncover and then enact planning, recruitment, participation, and data collection methods that are accessible, inclusive, and grounded in dignified language. Creating a safe method to partner with and to uncover diverse perspectives can be challenging, time consuming, and bespoke, as each person may require different supports for inclusion. However, this phase is of utmost importance, because it turns the vision phase into concrete application and practice.

Tasks for consideration during the uncover phase include:

1. Development of plain‐language documents for recruitment.
2. Embedding diversity into participant sample through inclusion criteria, methods selection, and form for participation, in balance with feasibility and rigour.
3. Conduct extensive user testing and pilot testing of data collection methods.
4. Offer flexible methods for participation, including options for a phone call, submission of various media, and alternate forms of engagement.
5. Manage access requirements by providing closed captions, easy‐to‐read English translation of documents, and interpreters as required (including Auslan) and communicating that there are funds and flexibility to support access.

#### 3.2.3 Discuss

Discuss describes the phase related to data analysis and was deliberately chosen by the working group to replace “analyse”, because data analysis should occur transparently and collaboratively with citizens with disability, rather than privately among rehabilitation researchers. Equitable distribution of input and power should be aspirational during discussion about data.

Tasks for consideration during the discuss phase include:

1. Discussing preliminary findings and emerging results with citizen science stakeholders and other community members in order to triangulate meaning in the context of diverse perspectives and experiences.
2. Coding indices, analysis methods, and mapping should be discussed collaboratively and be pre‐negotiated or changed to continue to privilege the voices of lived experience and ensure the normative dynamics of traditional research are rejected.
3. Ensure the breadth and depth of disability experience is engaged, beyond just the citizen scientists with disability on the research team.

#### 3.2.4 Iterative critical reflection

Iterative critical reflection is embedded in the framework to examine methods and ways of working that ensure transparency and inclusion. Although critical reflection comes after discuss in the framework, it is in fact iterative and can and should be conducted throughout the research process. The arrows flowing both ways across the graphic indicate the cyclical or iterative nature of the framework. However, following data analysis discussions, it is most important to reflect to ensure that both researchers and citizen scientist with disability have the opportunity to improve research practice, understand different perspectives, uncover previously unheard voices, and discuss methods and outputs prior to concluding research. Reflection includes examining what worked well and what may have presented incidental or explicit engagement barriers for citizen scientists with disability and research participants in order to improve practice or potentially collect additional data. The use of reflection journals may support identifying biases and bracketing those biases as much as possible. During reflection, engagement with the wider project team and stakeholders may be relevant. Reflection includes considering all the ways that the team authentically engages with citizens, including highlighting challenges and examples of good practice.

#### 3.2.5 Change

As often as possible, the working group agreed that research results should be used to drive change. The results of research using the Dignity Project Framework should be used to advocate for structural and systemic change at individual, community, and systems levels. Challenging norms and advocating for change is particularly important for rehabilitation research with citizen scientists with disability in order to increase the dignified experiences of the community.

Tasks to consider during this phase include:

1. Research findings should be disseminated using language that is best understood by the community, which may require plain language translation or abstracts and/or the use of easy‐to‐read English.
2. Reporting, publication, and dissemination should be done in ways that are transparent and accessible to the community from which citizen scientists with disability hail, which may include open‐access journal platforms.
3. Open and transparent dissemination increases community benefit and furthers the agenda of increasing citizen literacy in science.

## 4 DISCUSSION

The Dignity Project working group sessions and the resulting framework for dignified extreme citizen science identified many of the principles of inclusive research already developed in health research contexts. Principles of inclusion, accessibility, flexibility in research methods and approach, and negotiating reciprocity or remuneration are well discussed and outlined in health research generally (Cochrane Collaborative Consumer Network; Frankena et al., 2019) and occupational therapy research more specifically (Layton, 2014). Building on a foundation of inclusive research principles and principles of co‐design, the Dignity Project Framework for extreme citizen science extends citizen partnership and engagement in two critical ways: (1) an explicit defining and grounding of disability in a contemporary conceptualisation and (2) alignment with a specific method for conducting dignified research—extreme citizen science.

### 4.1 Promoting dignity through contemporary conceptualisations of disability

The working group recognised that citizens with disability encounter overt, covert, deliberate, and unconscious structural and attitudinal barriers that hinder engagement and partnership in research (de Melo‐Martin, 2010; Goodley & Runswick‐Cole, 2016; Meekosha & Shuttleworth, 2009; Pena et al., 2016; Robinson, 2017; Vehmas & Watson, 2014). For occupational therapy and rehabilitation research, ensuring dignified partnership for citizens with disability is especially important, as citizens may be sensitive or vulnerable to triggering experiences in re‐engaging with the health system, a system that lends itself to indignity. Disrupting and challenging attitudinal barriers can help to prevent dehumanisation and tokenistic views of disability, while enabling the proliferation of dignity for citizens with disability.

Explicitly grounding research in contemporary, human rights‐based understandings of disability in rehabilitation research works to disrupt attitudinal barriers by moving away from the medical model of disability, which is well entrenched in health service settings and health service research. Disability, according to the medical model, is caused by an individual's impairment or a supposed failing of the body (Berger & Lorenz, 2016; Hoksing, 2008). The medical model views impairment as something to be prevented or cured (Rioux & Valentine, 2006). Although the medical model may have its place in some aspects of occupational therapy and rehabilitation practice, it contributes to deficit and tokenistic consultation with citizens with disability in research. Contemporary understandings of disability recognise disability as a mismatch between person and environment but simultaneously recognise the everyday realities of impairment (United Nations, 2006). Although the language and conceptualisation of disability presented in this framework are currently contemporary, it is an ever‐accelerating and advancing field, making it even more critical for research teams looking to utilise this framework to engage their own citizens with disability to establish what is most current at that time.

### 4.2 Method specific alignment: The potential of extreme citizen science in health research

Although inclusive research principles and methods for engaging citizens with disability can succeed in uncovering and producing unique and different types of knowledge, the prevailing normative dynamics and values of institutional science often result in tokenistic consultation particularly as it pertains to marginalised and intersectional communities (Layton, 2014; Strasser et al., 2019). Frameworks for inclusive research often remain methodological unspecific, which enables application of principles into many contexts. However, the working group specifically aligned with extreme citizen science in the development of this framework, due to its potential to give make space for new views of knowledge production and its ability to privilege the voices of lived experience throughout the life cycle of research. For occupational therapy researchers, extreme citizen science can be flexible enough to accommodate the requirements of ethics committees regarding “vulnerable” populations while also ensuring that research addresses priorities of both funders and the disability community.

There is an important difference between citizens participating in ways that extend or validate science and citizens participating in research to generate “new knowledge structures and cognitive frameworks” (Irwin, 2015, p. 31). Within occupational therapy research and practice, citizens are often consulted for member checking, for triangulation, or to increase the validity of research. A research framework that embeds citizens with disability into the research team and throughout the research process ensures that they can contribute to the types of knowledge that are generated and decide what constitutes knowledge. In a clinical context, citizens with impairment often have insight into individualised adaptations and hacks for daily living, often outside of the typical scope of practice of occupational therapists. Consideration and value of the knowledge of lived experience could be used to improve practical outcomes. When partnering with citizens with disability in occupational therapy, it is critical to utilise method specific frameworks to realise dignity and equity in engagement.

Many of the principles of importance and tasks to consider throughout the framework are not just tasks to be carried out by occupational therapy researchers, but practitioners as well. Building a shared context and vision for therapy, working in flexible ways and meeting patients where they are at, can help to increase the dignified experience for patients. Communicating in plain language; in written, auditory, and visual formats; and frequently reflecting on personal biases in approach to care can improve the transparency and inclusion of occupational therapy. Dignity is important for care experiences, and by embedding the principles of importance and elements of this framework into clinical contexts, occupational therapists may increase the accessibility, inclusion, and dignified outcomes for their patients.

At its core, extreme citizen science can uncover new forms of knowledge, opening the way for academic researchers to be a necessary but not always dominant partner in exploring and understanding relationships between citizens and the world around them (Irwin, 2015). Traditionally, an elite group of “professional academics” or clinical researchers hold the power over what constitutes forms of accepted knowledge (English et al., 2018). Knowledge types include self, experiential, relational, social/political, temporal, professional, reflective, and interpretive (Morrow et al., 2012). Extreme citizen science values diverse types of knowledge and different epistemological understandings of knowledge production (Haklay, 2013). Achieving extreme citizen science that realises these aspirational principles of knowledge production is supported by the Dignity Project Framework, through creating and building mutual and shared context, and by ensuring citizen scientists with disability have a consequential and preferential say in how research is conducted, which can enable a dignified trajectory for rehabilitation and occupational therapy research (Chesser et al., 2020; Green, 2016; Hoksing, 2008; Layton, 2014; Strasser et al., 2019).

## 5 CONCLUSION

The imperative for occupational therapists to engage with consumers is increasing, both from national funding bodies and from within the growing range of best practice approaches to research. Whereas occupational therapy has a strong tradition of valuing person‐centred care and partnering with citizens to improve health outcomes, citizen engagement continues to emerge as a priority. The framework proposed in this article, while aiming to develop a framework that promoted dignified partnership with citizens with disability, is not without limitations. Although three citizens with disability were included in the working group and their perspectives were privileged in the creation of the framework, the authors recognise that three citizens do not represent the diversity of impairment and intersectionality. Implementation planning and feasibility studies were not yet developed for occupational therapy specific research, although clinical realities were considered during the working group sessions. Finally, the framework calls for embedding citizens with disability into the occupational and rehabilitation research life cycle from the outset in order to ensure that research is driven by the priorities most wanted by the community. However, the authors recognise that this could be difficult in practice, given the pressures of clinical research and limited research funding, which may not include large lead times or budgets for complete engagement prior to grant submission.

## CONFLICT OF INTEREST

The authors have no conflict of interest to declare.

## AUTHOR CONTRIBUTIONS

K.Ch. and A.D. formulated the research design and were responsible for the research processes, and the design of the framework. K.Ch. was the primary author of the manuscript with large contribution from C.E. E.K. and K.Co. were responsible for obtaining the funding and developing the principles of importance. All authors read and approved the final manuscript.

## Data Availability

xThe data that support the findings of this study are available from the corresponding author upon reasonable request.
